# Testicular shield for para-aortic radiotherapy and estimation of gonad doses

**DOI:** 10.4103/0971-6203.44477

**Published:** 2008

**Authors:** R. Ravichandran, J. P. Binukumar, S. Kannadhasan, M. H. Shariff, Kamal El Ghamrawy

**Affiliations:** Medical Physics Unit, National Oncology Center, Royal Hospital, Sultanate of Oman; 1 Department of Radiotherapy, National Oncology Center, Royal Hospital, Sultanate of Oman

**Keywords:** Clinical dosimetry, gonadal shield, para-aortic radiotherapy, stray radiations

## Abstract

For radiotherapy of para-aortic and abdominal regions in male patients, gonads are to be protected to receive less than 2% of the prescribed dose. A testicular shield was fabricated for abdominal radiotherapy with 15 MV X-rays ((Clinac 2300 CD, Varian AG) with low melting point alloy (Cerroband). The dimensions of the testicular shield were 6.5 cm diameter and 3.5 cm depth with 1.5 cm wall thickness. During treatment, this shield was held in position by a rectangular sponge and Styrofoam support. Phantom measurement was carried out with a humanoid phantom and a 0.6 cc ion chamber. The mean energy of the scattered photon was calculated for single scattering at selected distances from the beam edge and with different field dimensions. One patient received radiotherapy with an inverted Y field and gonad doses were estimated using calibrated thermo-luminescent detector (TLD) chips. Measured doses with the ion chamber were 7.1 and 3.5% of the mid-plane doses without a shield at 3 and 7.5 cm off-field respectively. These values decreased to 4.6 and 1.7% with the bottom shield alone, and to 1.7 and 0.8% with both bottom and top shields covering the ion chamber. The measured doses at the gonads during the patient’s treatment were 0.5–0.92% for the AP field (0.74 ± 0.17%, n = 5) and 0.5–1.2% for the PA field (0.88 ± 0.24%, n = 5). The dose received by the testis for the full course of treatment was 32 cGy (0.8%) for a total mid-plane dose of 40 Gy. The first-scatter energy estimated at the gonads is around 1.14 MeV for a primary beam of 15 MV for a long axis dimension of 37 cm of primary field. During the patient’s treatment, the estimated absorbed doses at the gonads were comparable with reported values in similar treatments. The testicular shield reported in this study is of light weight and could be used conveniently in treatments of abdominal fields.

## Introduction

Stray radiation doses to critical organs are of great concern during clinical radiotherapy. They arise from leakage from collimators and internal scatter from the primary irradiated volume.[1] The problem of peripheral doses is relatively more significant in beams of low megavoltages, especially in ^60^Co beams, as well as at lower distances from the beam edge, because of the higher scatter coefficient for side scatter.

Starkschall *et al*.[2] reported surface doses as high as 20 and 15% of the central axis dose, at a distance of 2 cm from the beam edge for ^60^Co photons and 15 MV beams. These doses were 8 and 5% for 10 and 12 cm distances reported for ^60^Co beams. Murthy *et al*.[3] confirmed peripheral doses of similar magnitudes at the gonads and eyes with ^60^Co and ^137^Cs beam qualities. For abdominal irradiations of male patients in the reproductive age groups, adequate shielding is necessary to preserve testicular functioning. Gonad doses > 50 cGy and cumulative doses > 200 cGy may lead to permanent injury.[1] Therefore, careful shielding of male gonads to < 2% of the prescribed dose is recommended to reduce the absorbed dose to this sensitive organ. Purdy *et al*.,[4] Fraass *et al*.,[5] and Bieri *et al*.[6] have outlined the efficacy of special testicular shields to achieve < 1.5% of mean doses prescribed to mid-plane. Although commercial designs are available for gonadal shields, many radiotherapy departments do not have these special shields readily available for immediate use. A testicle shield was locally fabricated in our mould room for the treatment of Seminoma Testis by Inverted Y portals. The protection offered by this custom-made shield was estimated by phantom measurements and during the treatment of the patient.

## Materials and Methods

### Fabrication of shield

**Figure 1:**
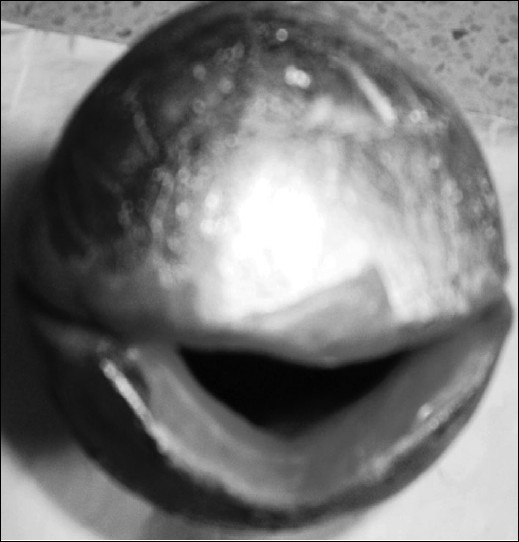
Front view of the top and bottom parts of the gonadal shield. The shield has a V cut for inserting the testis and to hold in position; wax coating is done on the inner surface of the shields.

Two semi-spherical half shields were fabricated with Low melting point alloy. An outer shell of a coconut of suitable dimensions was used as a positive. The hemispherical shield had an inner diameter of 6.5 cm, a maximal depth 3.5 cm at the center, and a wall thickness of 1.5 cm. In the top half shield, a V cut was made for the insertion of the scrotum and to place the top shield over it. A thin layer of dental wax coating was made for making the inner surface smooth and to avoid back-scattered electron dose from the shield [Figure 1].

### Phantom measurements

The abdominal part of a humanoid phantom was irradiated with an inverted Y field simulating the patient’s irradiation condition. Two sets of solid water sheets were kept to represent the thighs. The testis shield was kept in between the solid water sheets supported by commercial gel bolus sheets to provide scattering conditions. A 0.6 cc ion chamber (Farmer type) with a build-up cap was used to measure the stray radiation dose inside the shield. A dose 1 electrometer (Scanditronix Wellhofer) was used along with the ion chamber.

### Treatment of patient

One patient (M/28) received treatment for seminoma testis with 15 MV X-rays (Clinac 2300 CD, Varian). An inverted-Y field, AP/PA treatment with 37 cm length of the field was planned. Five fractions/week of a mid-plane dose of 40 Gy was prescribed in 20 fractions. During treatment, the shield was held in position by a rectangular sponge and styrofoam support kept in between thighs. A disposable plastic bag was used to keep the single testis inside the shield.

### Dose measurements in the patient

The patient’s dose to the gonads was measured using 4 mm × 4 mm TL chips (LiF: Mg). Doses were estimated as the mean values of doses to three TL detectors kept in mini-plastic packs. The TL detectors have the sensitivity to measure doses from 0.1 cGy to 10 Gy. The TL output was estimated using a TLD Reader (Model 4500 Bicron Harshaw). Measurements were made on five random days during both AP and PA treatments. To obtain the absorbed dose in cGy, the TL outputs were compared against the readings obtained for detectors exposed to a known dose at a depth of 5 cm in a tissue-equivalent phantom. Measured doses were expressed as percentages to the midline doses delivered to the central axis of the field.

### Calculation of mean scattered photon energy

**Figure 2:**
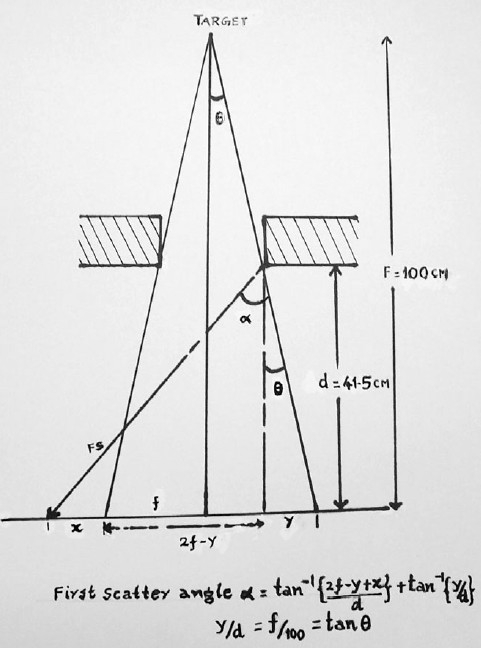
Schematic diagram of the geometry of the primary field with respect to the diaphragm of the linear accelerator and the patient’s entrance surface. The first scatter reaching a peripheral point at a distance ‘a’ from the field edge is shown. The scatter angle depends on FSD F, field size 2f, and ‘a’.

**Figure 3:**
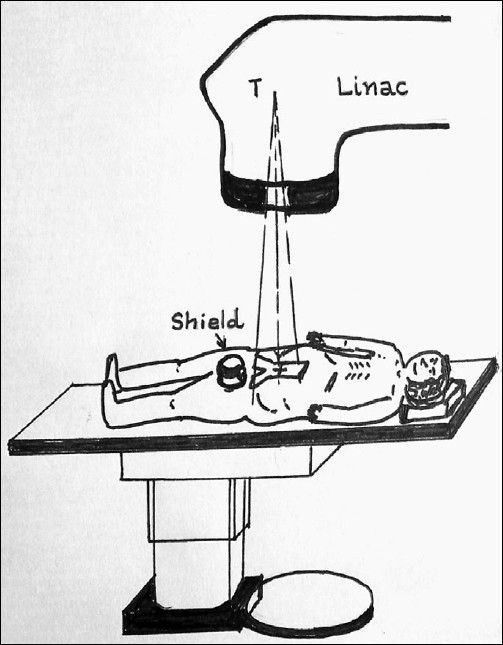
The relationship of the gonadal shield with respect to the inverted Y-shaped para-aortic field is shown in the AP view. The lower half of the shield reduces the scatter dose from the irradiated volume in the abdomen and the top half of the shield reduces the scatter and leakage radiations arising from the linac collimator and the machine.

Figures 2 and 3 show the geometry of the diaphragm, field width, and distance at which the first scatter reaches the position of the gonads. The energy of the scattered photon is calculated using the general formula of Compton Scattering (equation 1), substituting the value of scattering angle ‘α‘ [Figure 2] and the energy of the incident beam, hv. The mean energy of the incident beam is taken as 3 and 7.5 MeV for nominal photon energies of 6 and 15 MV respectively.

hν’=hν{1}{1+((hν/moc2)(1-cosα))}

## Results

The doses measured using the ion chamber in the phantom are shown in Table 1. Without the presence of the shield in position, the doses are 7.1 and 3.5% of the mid-plane dose at the central axis at 3 and 7.5 cm away from the proximal edge of the field, respectively, These doses have reduced to 4.6 and 1.7% with a bottom half shield and 1.7 and 0.8% with both bottom and upper shields present. Gonad doses inside the shield were 0.36 – 1.15 cGy for a mid-plane dose of 2Gy.

**Table 1:** Measured doses in phantom expressed as percentage of midplane dose delivered to patient

*Distance from field inferior border ‘x’cm*	*% Dose with no shield*	*% Dose Lower half shield alone*	*% Dose Lower + Upper half shields together*
3.0	7.1	4.6	1.7
7.5	3.5	1.7	0.8

Table 2 shows the TL measured doses which range from 0.5 to 0.92% of the mid-plane dose for the AP field (0.74 ± 0.17%, n = 5) and 0.5 to 1.2% of the mid-plane dose for the PA field (0.88 ± 0.24%, n = 5). The dose received by the testis for the full course of the treatment was 0.32 Gy (0.8%) for a total mid-plane dose of 40 Gy delivered by inverted Y fields. The energy of the scattered photons calculated at 7 and 14 cm from the edge of the field, for 6 and 15 MV primary photons are shown in Table 3. It can be seen that 15 MV X-rays gives rise to greater scatter energy. The effect of the scattering angle ‘α’ with the long axis of the field 20 cm, 30cm are also seen For a field dimension of 37 cm, a 15 MV photon beam gave a scatter energy of 1140 KeV at a distance of 7 cm at the position of the gonad. The 1.5 cm thickness of the low melting point alloy shield was therefore able to provide shielding to address the scattered 1140 KeV photon during actual treatment.

**Table 2:** Measured gonad doses by TLD during treatment on different days

*Field*	*Estimated gonad doses in cGy for midplane dose 100cGy*					*Mean dose to gonads cGy*

	*1*	*2*	*3*	*4*	*5*	
AP	0.51	0.87	0.60	0.92	0.78	0.74 ± 0.17
PA	0.54	0.83	0.87	1.21	0.93	0.88 ± 0.24

**Table 3:** Energy of the first scatter reaching the point proximal to the radiation field

*Long axis of primary field cm*	*Scatter photon energy for 6 MV KeV*		*Scatter photon energy for 15 MV KeV*	
	*X = 7 cm*	*x = 14 cm*	*X = 7 cm*	*X = 14 cm*
20	1474	1215	2090	1605
37	929	822	1140	984

## Discussion

This report highlights our experience in designing and fabricating a prototype gonadal shield for inverted Y field radiotherapy. Compared to earlier reported versions of shields for such applications, our gonadal shield made out of low melting point alloy is less heavy and is convenient for clinical use. The spherical shape of the shield has very little weight compared to earlier types which were box-shaped. As the scattered photons from the diaphragm will be in all planes, the spherical shape of the gonad shield is able to provide adequate shielding to the testis in all the directions. The magnitude of the doses measured here both by phantom and patient measurements in the custom-made shields are comparable with the values reported in literature[3–6] [Table 4].

**Table 4:** Stray radiation doses at the testes from different studies

*No*	*Reports from*	*Energy of photon beam*	*% Testicular Dose from Without Shield*	*AP field With Shield*	*% Testicular Dose from Without Shield*	*PA field With Shield*
1	Murthy *et al* (3)	Co-60
2	Purdy *et al* (4)	Not mentioned	4 – 6%	1.5–2.5%	-----	-----
3	Fraass *et al* (5)	10MV	2.00%	0.40%	2.5%	0.5%
4	Bieri *et al* (6)	18MV	1.95%	0.74%	0.93%	0.33%

All the above studies have shown that providing shielding near the collimator (at the tray level) is not efficient to reduce gonadal doses; therefore, additional shields surrounding the testes have been recommended. These studies have confirmed that as the distance of the gonads from the lower edge of the primary field increases, the gonadal dose also decreases. A study by Fraass *et al* indicated that the depth of measurement inside the shield has little effect on the measured doses. These authors have kept TL detectors on the thighs to represent doses without a shield, whereas in the present work, we kept the TL detector on the outer surface of the spherical shield to represent doses without a shield. Results reported by Fraass and co-workers showing little effect on the measured dose values with and without build-up, clearly outlined that low-energy photons are encountered at the position of the gonads. This indicates the adequacy of 1.5 cm thickness of shield for the gonads, although the primary beam is 15 MV. It can be seen in Table 1, that at a distance of 7.5 cm from the field border, the presently designed gonad shield has provided a shielding factor of 3.5/0.8 = 4.375. This factor is more than the shielding factor of 2.5–2.8 seen at distances greater than 15 cm such as for the gonadal shield reported by Fraass *et al*.[5] We have used a 4 mm build-up cap with the 0.6 cc chamber, which would have included some differences in measurement at the reduced quality of the stray radiation encountered, but it was earlier reported that build-up does not have significance. Columns 3 and 4 in Table 1 indicate that the bottom half shield reduced internal scatter and that the top half shield prevented head leakage and collimator scatter from reaching the testis. The linac head radiation leakage for 15 MV is about 50 mGy/h in our linac. At the patient’s dose delivery of 300 MU/min, the leakage reaching the gonads from this per field will be about 1.5 mGy, which is in agreement with the reported values in Table 1.

A previous report with ^60^Co beam has been included in Table 4 to show the need for such a study in these beam qualities. Murthy *et al*.[3] have performed their study with the objective of studying the effect of field size, the effect of elimination of low energy photons at very near distances from the field edges within 1–10 cm, the effect of penumbra trimmers, and the effect of back scattering medium. Therefore, the shielding efficacy of such gonad shields in the application of low megavoltage beams like ^60^Co beams could be investigated by a separate study. The present work has highlighted clinical dose measurements to confirm that doses as low as 0.8% of the prescribed dose can be achieved. The contribution of the dose at the testis is mainly due to head leakage and internal scatter, and the spherical shield could give adequate shielding. Although the work carried out is an old concept in conventional radiotherapy, we have reported the technical details with a view that it is of clinical interest in many institutions where such special shields are not available, and they have to resort to the use of nonoptimal solutions.
